# An Indoor Location-Based Control System Using Bluetooth Beacons for IoT Systems

**DOI:** 10.3390/s17122917

**Published:** 2017-12-19

**Authors:** Jun-Ho Huh, Kyungryong Seo

**Affiliations:** 1 Department of Software, Catholic University of Pusan, Geumjeong-gu, 57 Oryundae-ro, Busan 46252, Korea; 72networks@pukyong.ac.kr or 72networks@cup.ac.kr; 2 Department of Computer Engineering, Pukyong National University, Busan 48513, Korea

**Keywords:** Bluetooth beacon, Java Android implementation, Artificial Intelligence, Python implementation, IoT, indoor localization, control system

## Abstract

The indoor location-based control system estimates the indoor position of a user to provide the service he/she requires. The major elements involved in the system are the localization server, service-provision client, user application positioning technology. The localization server controls access of terminal devices (e.g., Smart Phones and other wireless devices) to determine their locations within a specified space first and then the service-provision client initiates required services such as indoor navigation and monitoring/surveillance. The user application provides necessary data to let the server to localize the devices or allow the user to receive various services from the client. The major technological elements involved in this system are indoor space partition method, Bluetooth 4.0, RSSI (Received Signal Strength Indication) and trilateration. The system also employs the BLE communication technology when determining the position of the user in an indoor space. The position information obtained is then used to control a specific device(s). These technologies are fundamental in achieving a “Smart Living”. An indoor location-based control system that provides services by estimating user’s indoor locations has been implemented in this study (First scenario). The algorithm introduced in this study (Second scenario) is effective in extracting valid samples from the RSSI dataset but has it has some drawbacks as well. Although we used a range-average algorithm that measures the shortest distance, there are some limitations because the measurement results depend on the sample size and the sample efficiency depends on sampling speeds and environmental changes. However, the Bluetooth system can be implemented at a relatively low cost so that once the problem of precision is solved, it can be applied to various fields.

## 1. Introduction

The new trend in the (MEMS) Micro-Electro-Mechanical Systems has improved the ability of automatically recording, processing and transmitting information through different infrastructures. Also, many new generation devices, sensors actuators such as RFID/NFC/Bluetooth equipment, (WSN) Wireless Sensor Networks, embedded systems have created new markets. For the coordination between different architectures having varied services and applications, a research on their theoretical foundations, security issues, programming potential energy efficiency/management is required.

Also, people always dreamt about a smart system that acts like an AI (Artificial Intelligence)-based butler Jarvis in the movie series, Iron Man. Although this was regarded as a fantastic but a fanciful story in the past, it is not far from becoming a reality as the AI being like Alpha-Go [1] has started making an appearance while the era of IoT (Internet of Things) is emerging rapidly. The system that recognizes the location of all those “Things” making access automatically in real-time can do much smarter work.

The most typical way of obtaining location information is to use the GPS (Global Positioning System) service [2]. This system has improved people’s quality of life by providing their locations through devices such as smartphones and portable navigators which also provide the distance or the route information to the target point thereby reducing the cost and time. Although the GPS technology is powerful and quite useful, its functions cannot be performed properly in the spaces within buildings, underground, or tunnels. Because of such a disadvantage, the necessity of an adequate indoor location-based service has arisen recently and many R&D’s are being actively carried out.

In this paper, Indoor Localization will be performed by deploying the BLE under the WLAN (Wireless LAN) environment. The BLE technology allows a very low-energy operation and is being used widely.

This paper is an extended version of the proceeding [3] presented at one of the international conferences. A number of additions have been made in the Related Study section along with the Location-Based Control theory. By describing the test method and the UI (User Interface) more clearly, this improved version will facilitate the understanding of the proposed system.

We have developed a system that can determine the current location of a user as soon as he/she enters a closed space (indoor) and control household devices based on location information.

## 2. Related Research

### 2.1. Localization Technologies

Most of localization technologies involve spatial partitioning, RSSI, trilateration and CG (Center of Gravity). The spatial partitioning defines a basic unit of a spatial unit and determines the locations where beacons to be deployed. The RSSI is a numerical indicator of the signal power received by the wireless receiver. This is used to calculate the distance to the location from which the signal has been sent.

#### 2.1.1. Spatial Partitioning

The indoor space is divided into a multiple number of hexagonal basic unit spaces. The size of a unit varies depending on the size of indoor space. The beacons are deployed at each corner and center of the unit [3,4,5,6]. Therefore, a total of seven location beacons are deployed within a single basic unit space to perform trilateration by selecting four beacons transmitting strong signals. All the beacon locations in the space will be relative. When the indoor space is large, many basic unit spaces can exist and the system can compute the indoor absolute coordinates easily.

Geometrically, the largest area can be obtained from a circle when the circumference or the length is fixed but when there are a multiple number of circles to be connected, the empty spaces will be created so that it is not possible to utilize the spaces effectively.

On the other hand, the regular hexagons do not allow any gaps between them as their sides fit perfectly to each other. Although it is true that other shapes like triangles or rectangles have the same characteristic, much more materials are required to form the wall surface while generating a narrower space.

The hexagonal shape is the most economical structure that provides the largest space with the least materials in addition to being a stable structure that evenly distributes forces so that it is used in the construction engineering.

#### 2.1.2. Basic Unit Space and Deployment of Location Beacons

**Figure 1:**
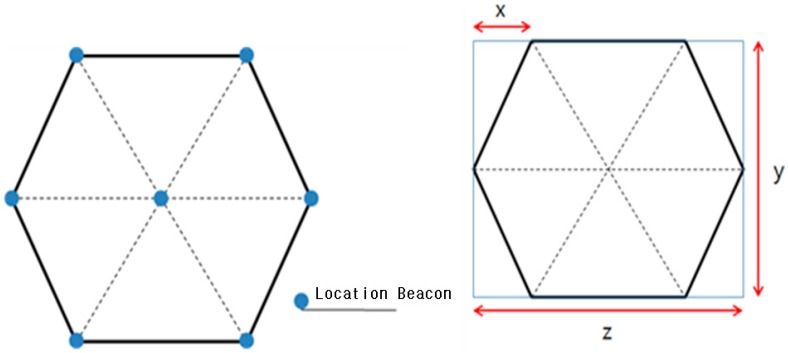
A basic unit space with beacons.

A basic unit space takes a hexagonal form. For convenience, Figure 1 represents it as a regular hexagon. A total of seven location beacons are deployed at each corner and the center of the basic unit space. As the space is hexagonal, each beacon’s location can be estimated by using the lengths w, y, z.

#### 2.1.3. Space Extension

**Figure 2:**
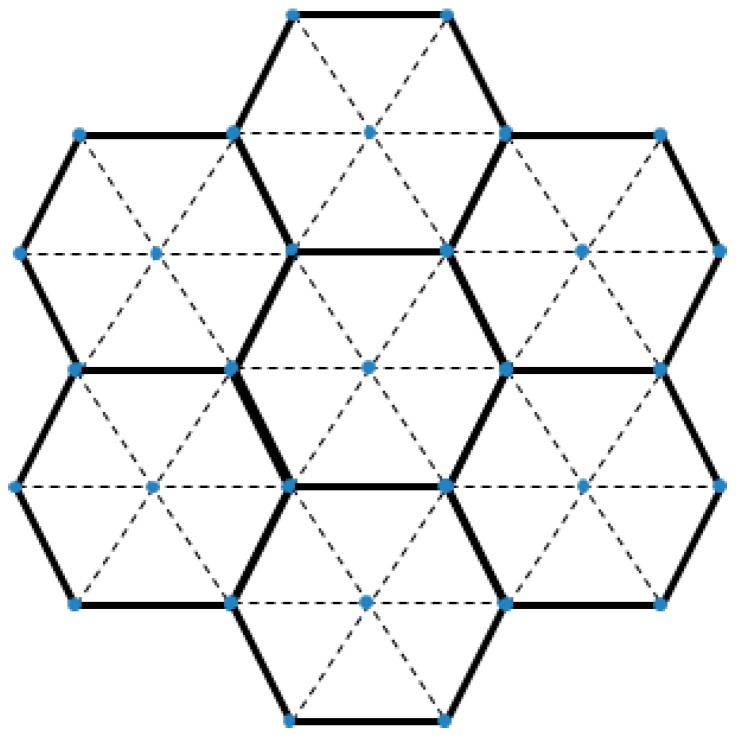
Indoor space partitioning for a large space.

If the indoor space is very large, localization will not be easy with just a single basic unit space. It is convenient to extend the space as the basic unit spaces are hexagonal. Figure 2 shows the partitioning of a large space.

Two problems could arise when the indoor space is divided into a multiple number of basic unit spaces. First, the basic unit spaces share deployed location beacons and the second, the locations within the basic unit space are of relative coordinates. There are two ways to solve these problems. The first is to let the user application maintain only the identification number of the basic unit space where location beacons transmits signals. However, the system has to find out in which basic unit space the beacons are deployed when the application starts to act. In this case, a trilateration that extends beyond a single basic unit space can be performed. The second solution is creating an absolute coordinates table for each basic unit space separately. By querying the table using an index in order to obtain the relative coordinates, an absolute coordinate can be determined.

### 2.2. RSSI (Received Signal Strength Indication)

The RSSI is a numerical indicator of the signal power received by wireless receiver and its unit is dBm [7,8,9,10,11]. In this study, RSSIs are used to measure the distances and for the wireless signal communications, Bluetooth 4.0 was used. As wireless signals become weaker in proportion to the distances they travel, one can measure a specific distance by using a path-loss model.

#### 2.2.1. Characteristics of Wireless Signals and Bluetooth 4.0

In general, wireless signals have three negative characteristics: signal strength attenuation, signal interference and multipath propagation. The attenuation occurs when electromagnetic radiation becomes weaker as they pass through objects. Such a phenomenon also occurs in a vacuum. Thus, the strength of a wireless signal is inversely proportional to the distance between transmitter and receiver. This is called a path-loss. Signal interference refers to the phenomenon where the transmission data cannot be extracted at the receiver side due to the interferences between the wireless signals transmitted with an identical frequency bandwidth. Finally, multipath propagation refers to the phenomenon where wireless signals reach the receiver individually through multiple paths of different distances as they collide with some objects [3].

In this task, we attempt to measure the distance between transmitter and receiver by using a path-loss model. In an ideal indoor space where there are no signal-interrupting objects, the first signal arriving at the receiver can be described to have taken a direct path between two devices. However, in reality, the signals arrive at their final destinations taking much longer routes after colliding with furniture or moving people within the indoor space. Considering this factor, we installed the location beacons on the ceiling. As the measurements obtained through Indoor Localization always have some errors due to multipath propagation, we try to reduce the errors by setting the COG (Center of Gravity) of each candidate location as the final location.

The reason for selecting Bluetooth as a wireless link is that it is less costly and requires low power. Bluetooth 4.0 technology consumes much lesser power than the previous ones and such a low-energy consumption method is called BLE [7,8,9,10,11]. Many location beacons are needed for the system as the indoor spaces are partitioned into multiple hexagonal basic unit spaces. Using the low-cost Bluetooth modules can reduce the system implementation costs.

#### 2.2.2. A Path-Loss Model and Distance

Signal strength according to distance can be represented by (1) (a path-loss model), in which *n* is a signal attenuation constant, *d* is the distance between transmitter and receiver and *Tx* is the strength of the transmission signal measured at a distance of one meter from the transmitter.

*RSSI*[*dBm*] = −(10*nlog*_10_*d* − *Tx*) (1)

Since the interior of each indoor space can be different, the signal attenuation constant *n* is determined through experiment and the signal strength can be represented with a RSSI value. 

That is, the transmitter has a specific *Tx* value set by the manufacturer so that *d* can be calculated thereby *n* can be also calculated using (1). Below Table 1 shows the *n*-values obtained from different distances. Calculating the distance *d* based on (2) with a constant *n*, following equation can be established.

(2) $$d[m]=10^{\frac{RSSI-Tx}{-10n}}$$

The detailed description of the experimental space can be found in Section 3.2.2.

#### 2.2.3. Other Indoor Localization Technologies

The lower transmission power of BTLE/BLE also contributes to the better performance of localization because it can reduce the multipath effect in some scenarios. Since the sensitivity of receivers is almost the same for both BTLE and Wi-Fi devices, in extreme cases the receiver can only hear the most powerful signal component, e.g., the line-of-sight signal, while all others are filtered out [12]. Also, Ramsey Faragher et al. demonstrated that significant positioning improvement over Wi-Fi is possible using even a relatively sparse deployment of beacons once the characteristics of BLE signals are accounted for [13]. Suining He et al. implemented Tilejunction, simulation and experimental measurements, showing that it outperforms other recent state-of-the-art approaches (e.g., RADAR, KL-divergence, etc.) with significantly lower localization error (often by more than 30 percent) [14]. Meanwhile, BLE technology provides the variables that can be used to predict the position information. One of those variables is the received signal strength indicator (RSSI), which may give an estimate of the flight distance of the signal from the beacon to the receiver [15,16]. Neburka et al. [15,16,17], after extensively analyzing the performance of BLE technology in indoor environments, show that it is a promising technique for indoor positioning, even though the RSSI values are inaccurate and highly dependent on the BLE module used. Its new abilities—such as durability, mobility and high reaction time—have led to Bluetooth BLE technology replacing Wi-Fi for positioning purposes.

There also exit some infrastructure or device-based localization technologies as well. The former uses WLAN, UWB (Ultra Wide Band), RFID (Radio Frequency Identification) and IR (Infrared) technologies, while the latter employs the inertial sensors of mobile devices such as accelerometers or gyroscopes [18,19,20].

The WLAN-based systems normally measure the distance based on the wireless signal strength or TOF (Time of Flight) in the network. The UWB-based systems use a wireless technology that transmits a large amount of digital data through a wide-spectrum frequency band. This consumes relatively more power than Bluetooth system and costs more but has a lesser background noise and wider transmission distance [21,22,23,24,25]. Meanwhile, the RFID-based system is uses a technology that identifies objects or people by using radio frequencies. This may not be able to locate indoor positions accurately but can determine their proximity. Finally, IR-based system is employs a technology that estimates the position by obtaining the 3D-depth information by using TOF (infrared) with a IR-Depth camera. A 3D map can be produced by mapping the depth information onto the 2D image taken with a regular camera.

### 2.3. Trilateration 

**Figure 3:**
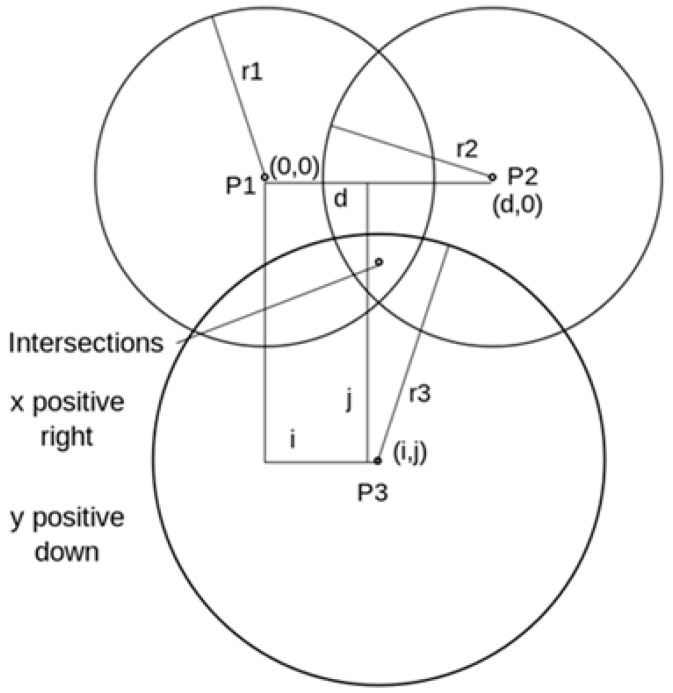
An example of trilateration.

Trilateration is a technique that calculates the intersecting point of three circles when information concerning the three circles or a sphere is given. Figure 3 shows the intersecting point (x, y) when the center points (P1, P2, P3) of three circles and the radii (r1, r2, r3) are already known [21].

In Section 2.2, it was possible to determine the distance between transmitter (location beacon) and receiver (user device) through the path-loss model of a certain wireless signal.

#### 2.3.1. Trilateration

Following equations represents the three circles shown in Figure 3.

(3) $$r_{1}^{2}=x^{2}+y^{2}$$

$$r_{2}^{2}={\left(x-d\right)}^{2}+y^{2}$$

$$r_{3}^{2}={\left(x-i\right)}^{2}+{\left(y-j\right)}^{2}$$

Combining the equations in (3), we were able to find the intersecting point (*x*, *y*) as the location beacons were installed on the ceiling to minimize the interferences during signal propagation and they did not exist on the same plane where the user device exists.

Thus, (3) should be modified to create a 3D space.

(4) $$x=\frac{r_{1}^{2}-r_{2}^{2}+d^{2}}{2d}$$

$$y=\frac{r_{1}^{2}-r_{3}^{2}+i^{2}+j^{2}}{2j}-\frac{i}{j}x$$

(5) $$r_{1}^{2}=x^{2}+y^{2}+z^{2}$$

$$r_{2}^{2}={\left(x-d\right)}^{2}+y^{2}+z^{2}$$

$$r_{3}^{2}={\left(x-i\right)}^{2}+{\left(y-j\right)}^{2}+z^{2}$$

$$z=\pm \sqrt{r_{1}^{2}-x^{2}-y^{2}}$$

In performing trilateration, the number of intersecting points must be greater than one when there are two or more circles or spheres. That is, the condition $d-r_{1}<r_{2}<d+r_{1}$ must be satisfied. As discussed in Section 2.2.1, the multipath propagation of wireless signal can occur due to some obstacles on the path. The system disregards the RSSI value of a certain signal when the condition of trilateration has not been satisfied.

#### 2.3.2. Selecting the Target Points for Trilateration

**Figure 4:**
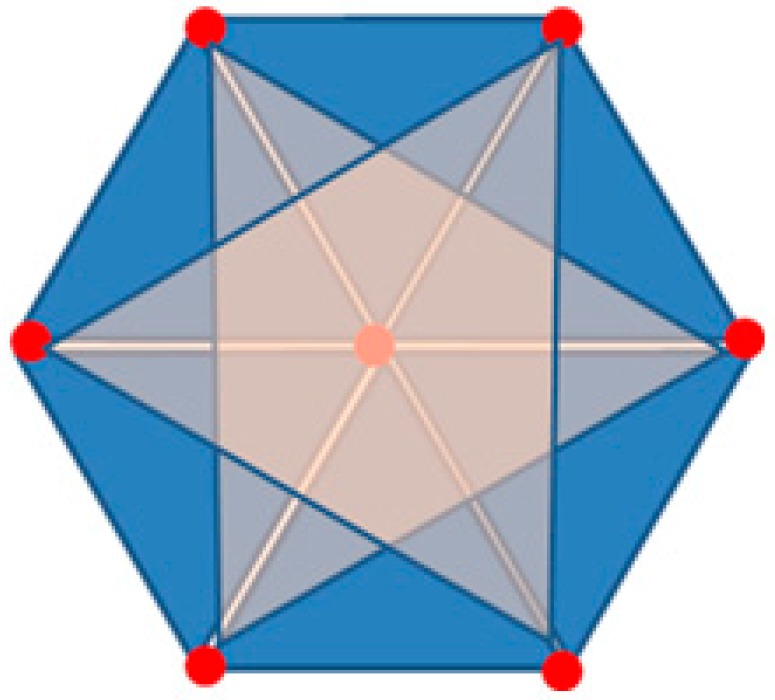
Selecting the target points for trilateration.

The user device delivers the RSSI values obtained from the seven location beacons in a basic unit space to the system which will then calculate the distance by using the values based on the path-loss model. Now the system performs trilateration knowing the radius and center of each location beacon, the possible combinations of selecting three location beacons among seven of them is 7C3 so that there are 35 combinations. However, the system will not deal with all of them but will instead perform eight trilaterations for the six smaller triangles and the two larger triangles, as shown in Figure 4. 

### 2.4. Center of Gravity (COG)

**Figure 5:**
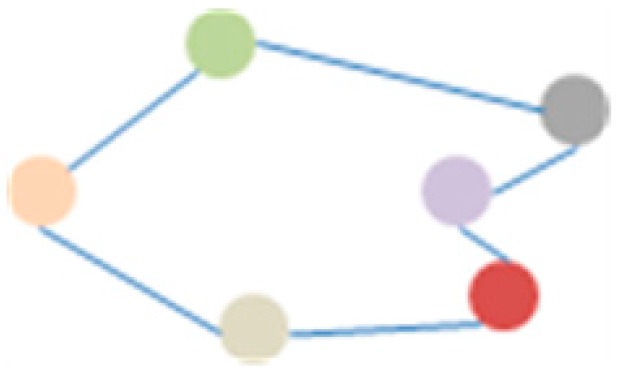
The COG of a Polygon.

A shape shown in Figure 5 was produced with six candidate locations after performing a trilateration.

As shown in Figure 5, the COG of polygon has been determined as a final location after connecting the candidate locations calculated through trilateration. Saying that the location of each candidate is $\left(x_{i}-y_{i}\right)$, the COG of the polygon (x−y) can be represented as:

(6) $$S=\frac{1}{2}\sum_{i=0}^{N-1}\left(x_{i}y_{i+1}-x_{i+1}y_{i}\right)$$

$$x=\frac{1}{6S}\sum_{i=0}^{N-1}\left(x_{i}+x_{i+1}\right)\left(x_{i}y_{i+1}-x_{i}y_{i}\right)$$

$$y=\frac{1}{6S}\sum_{i=0}^{N-1}\left(y_{i}+y_{i+1}\right)\left(x_{i}y_{i+1}-x_{i}y_{i}\right)$$

## 3. Indoor Location-Based Control System

**Figure 6:**
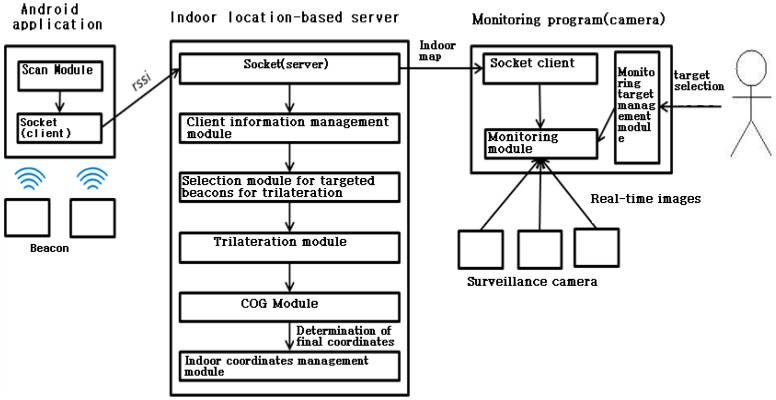
The schematic of indoor location-based control system.

The indoor location-based control system consists of a localization server, service-provision client, user application and positioning technology. The last element has been discussed in Chapter 2 so that this chapter explains how the rest of elements operate based on the positioning technology. Figure 6 shows the schematic of indoor location-based control system [3].

### 3.1. Localization Server

The localization server manages the accesses made by the client’s devices such as smartphone or other wireless devices, estimates their indoor locations and maintains the indoor map. Python v2.7 was used for programming and the server transmits acknowledgements when performing socket communications.

#### 3.1.1. Operation Process

**Figure 7:**
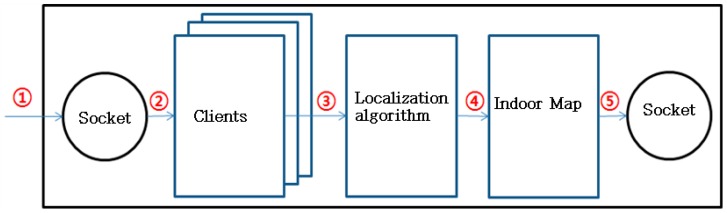
The operation process of the localization server.

Figure 7 shows the operation process of the localization server:

① The client attempting to access the server exchanges information via socket communications with it. The communication commands will be explained in Section 3.1.2. The client can be a mobile phone or a service program.
② The server distinguishes individual devices with their unique IDs and generates an object(s) that represents client’s device(s).
③ Using the localization technology described in Chapter 2, indoor position is calculated with the RSSI values periodically delivered.
④ Maintain the table containing the indoor location information of each device connected to the server.
⑤ Provide an indoor map to the service-provision client which will be discussed in Section 3.2.

#### 3.1.2. Communication Commands

There are respective communication commands for client and server. First, the commands used by the clients are listed in Table 2.

Each unique identifier is a 4-digit number between 0000 and 9999 and is generated by a random selection function for the client. The numbers from 0000 to 0999 are used as the unique identifiers for the service-provision client whereas the numbers from 1000 to 9999 are used for the user devices. The RSSI “minor” is one of the information that identifies the location beacon. The location beacons are classified as YYID (Universally unique identifier), for both major and minor. In practice, the beacons within the same building are distinguished based on the minors, all of which has a 2-byte value from 1 to 65,535. The RSSI value is a whole number. Table 3 shows the commands used by the server.

If the sever interprets the IDEN command properly, then the corresponding acknowledgement response will be IDEN OK. IDEN OVERLAP means that the registered unique ID is already being connected to the server. The coordinates (x, y) in the LIST shows the indoor location of a device relevant to the ID. A rough domain is represented with “area” for convenience and it is a positive integer.

### 3.2. Service-Provision Client

A variety of services including indoor navigation, monitoring and surveillance can be provided with the indoor map created by the localization server. For this task, a monitoring/surveillance program has been implemented. The program was developed with Java JRE 1.8 using Java Swing.

#### 3.2.1. Operation Process of Camera-based Monitoring Program

**Figure 8:**
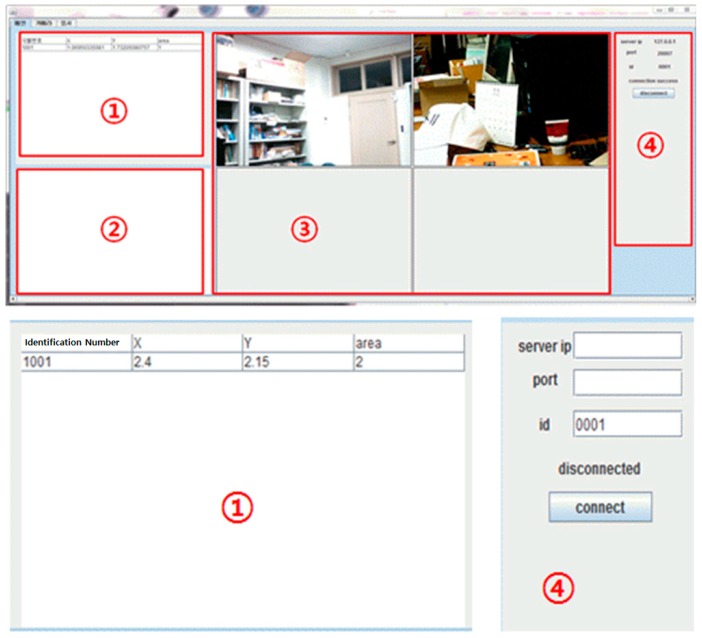
A basic screen of the camera-based monitoring program.

As the conceptual system diagram in Figure 6, the camera-based monitoring program acquires an indoor map by performing socket communications with the server, during which the LIST commands are transmitted to the server every five seconds by generating a communication thread each time. The monitoring module shows the images taken by the surveillance camera in real time and displays map’s information in the table view. The program user can track the location of a certain mobile phone through this table view. The program generates two threads to update the screen. One of them is to update the real-time images and the other is to update the domains ① and ② in Figure 8. This is to process other demands from the user more quickly. 

#### 3.2.2. User Interface

**Figure 9:**
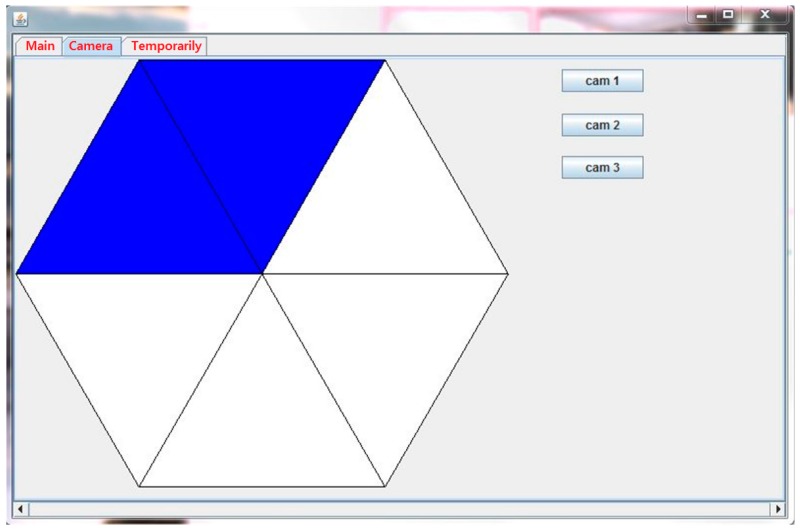
Implementation of the screen settings for camera-based monitoring program.

The UI (User Interface) for the camera-based monitoring program is shown in Figure 8. First, in Domain ①, the indoor map is presented with a table view where a row of identifiers (x, y, area) will be shown. Domain ② represents the indoor location of a mobile phone graphically. Domain ③ displays an image taken by the linked camera and finally, Domain ④ is the area that deals with the operations related to the access with the localization server. Here, Domain ① and ② are updated periodically every 0.546 s. As in Figure 9, the surveillance domain can be adjusted when starting the program.

### 3.3. User Application

The user application implemented in this study performs socket communications with the server to assume the role of sending beacon’s RSSI. This is an Android app and its minimum requirement is above API level 21 (Marshmallow). If the mobile phone is not the latest model, it will take quite a long time to scan Bluetooth signals.

#### Operation Process

**Figure 10:**
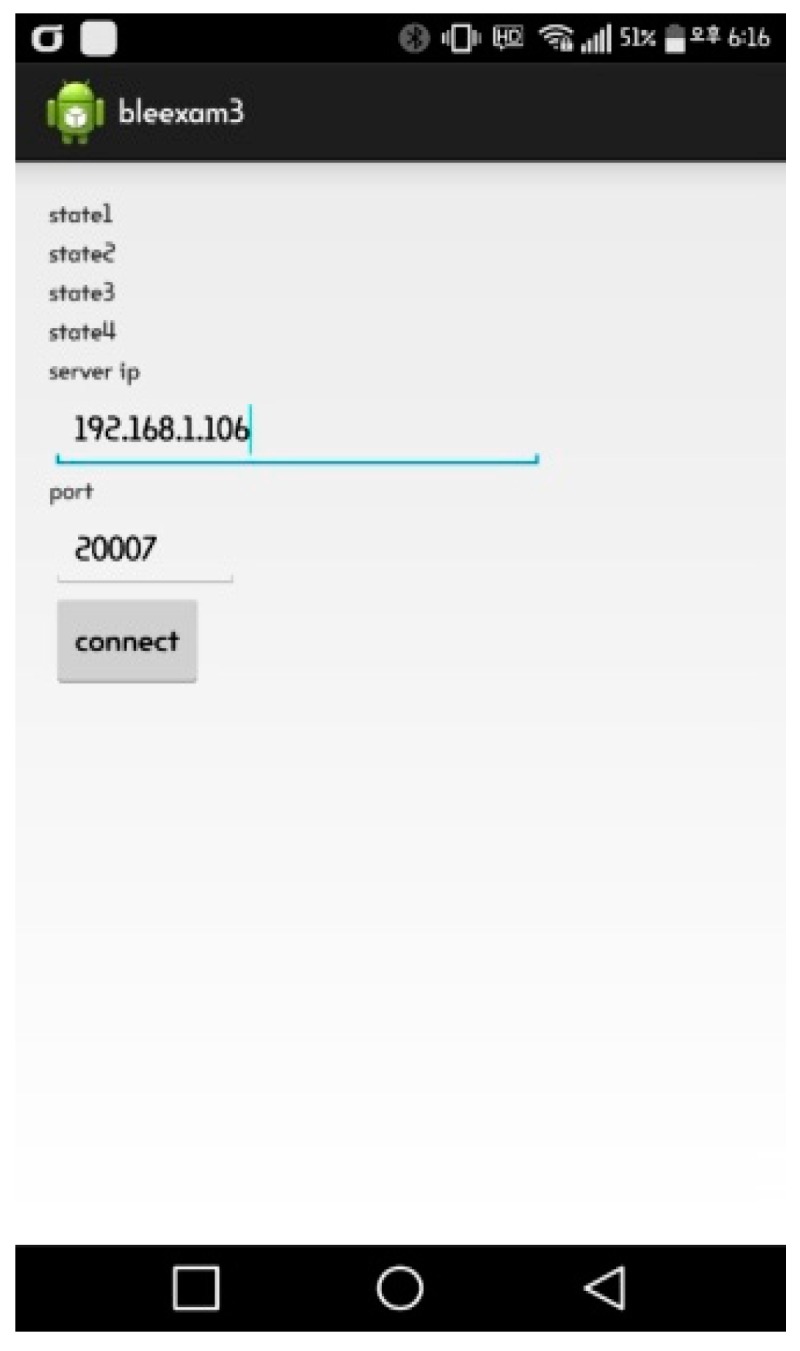
Execution of user application (Scenario 1).

The Android application in Figure 10 accesses the server to scan Bluetooth signals. Its transfer thread transmits the RSSI of a scanned Bluetooth signal to the server every 0.5 s following the RSSI command. Due to the multipath propagation, several signals can be sensed by the scanning module on a different timetable even though they are originating from the same location. To solve this problem, only the largest RSSI in a 0.5 s transmission cycle will be recorded and the process will be restarted.

The screen in Figure 10 shows four items that indicate the operational state of the application along with a connection button with the server. The connection will be established by entering the server IP and port numbers first and pushing the connect button afterwards.

## 4. Results of Experiment and Considerations

### 4.1. Scenario 1 Test Environment 

The experiments were conducted in a school lab. The size of the laboratory and a description of it are provided in Section 4.1.1, followed by the description of the equipment used for the experiments in Section 4.1.2.

#### 4.1.1. Testing Space

**Figure 11:**
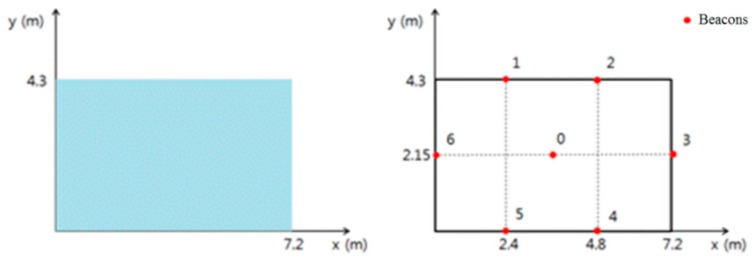
The coordinate system and location beacons deployment plot of the testing space.

The experiments were carried out in Room No. 2110, Nuri Hall, Pukyung National University. The size of the testing space is shown on the left picture in Figure 11, consisting of 7.2 m and 4 m in length and breadth, respectively. This is a narrow space that represents a single basic unit space described in Section 2.1.1. The signal attenuation constant has been set at 4.610 for the distance of 3 m, as in Table 1.

**Figure 12:**
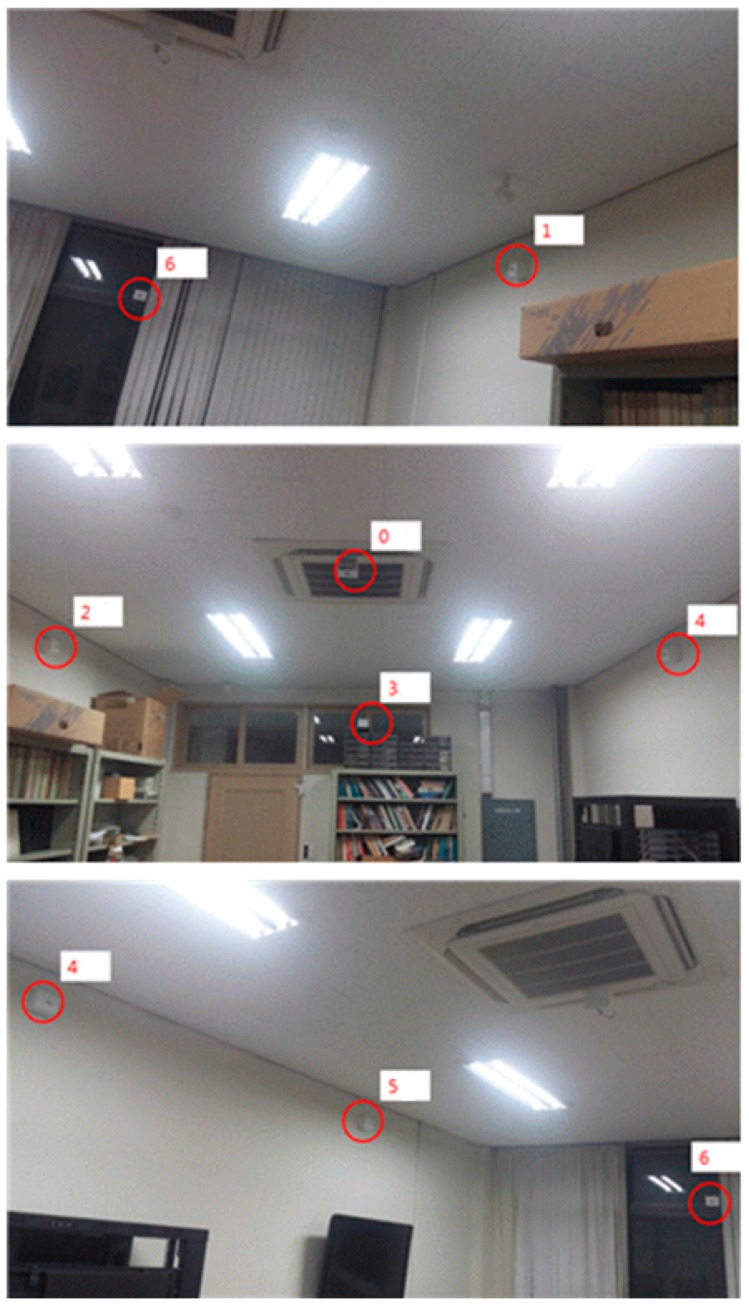
Actual deployment status of location beacons in the testing space.

**Figure 13:**
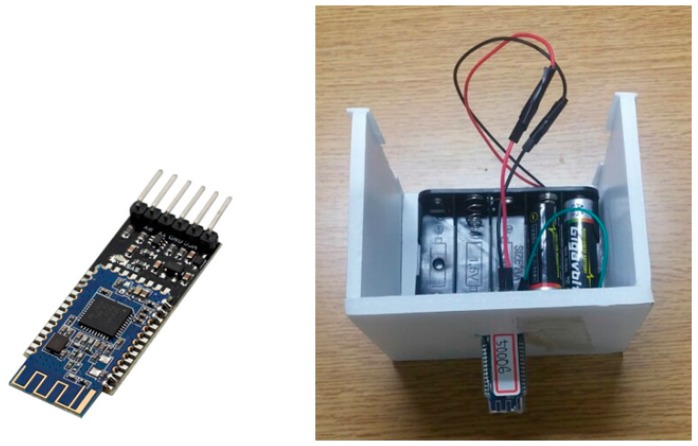
Bluetooth module (HM-10) and location beacon experiment tool (Using Scenario 1).

Figure 12 below is a picture of the deployment status of location beacons. The number of beacons in Figure 11 and Figure 12 is the same and they have been installed on a ceiling to minimize signal interruptions. The structural appearance is shown in Figure 13.

#### 4.1.2. Testing Equipment

How and with which equipment each system component described in Figure 12 was operated is being explained in Table 4.

The HM-10 Bluetooth module is shown on the left photo in below Figure 13.

It needs to go through a series of process to prepare a HM-10 module as a beacon. First, link Arduino UNO R3 with a computer and upload communication program to enable Bluetooth serial communications. Then, connect R3 with the HM-10 module. According to the data sheet of HM-10, HM-10 can operate location beacons by using the commands listed in Table 5. The rated voltage of HM-10 is set at level from 2.7 V to 3.3 V.

#### 4.1.3. Experiments

Check whether the system’s major elements (i.e., user application, localization server and camera-based monitoring client) have properly operated as expected in Chapter 3. Then, observe the accuracy of the localization server.

Deploy location beacons as shown in Figure 12 and proceed with experiments using the equipment listed in Table 4. After completing preparatory work, execute the user application to connect with the server. Test the system for about 135 s and document the indoor map delivered by the camera-based monitoring client by using the file output function. The indoor map is updated and documented every 0.546 s. Based on the documented data, observe the accuracy of localization.

#### 4.1.4. Experiment Results and Performance Evaluation

**Figure 14:**
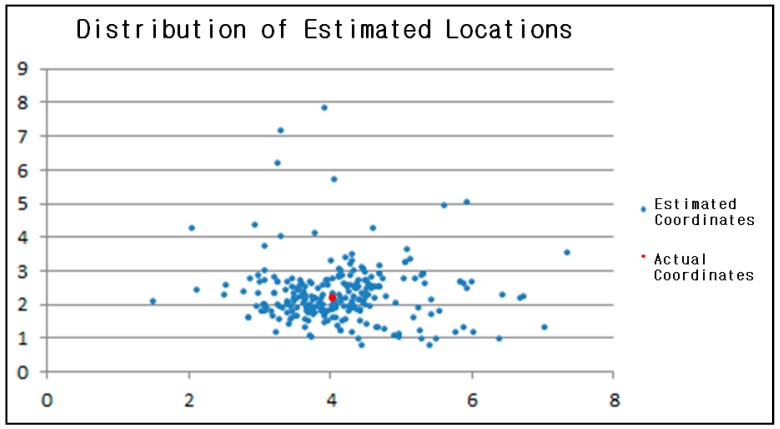
Distribution graph for the estimated locations.

The graphical results are shown in Figure 14. At the actual coordinates (4, 2.15), 199 out of 269 estimated locations fell within the margin of error when the error range was 1m so that it is possible to say that the accuracy of the localization server is approximately 74%.

The results in Figure 14 were obtained from the situation where there are almost no obstacles and only one person exists within the testing space. The accuracy will be lower in an indoor environment that has many obstacles. The most important factor for the indoor location-based systems is the accuracy of localization. Thus, additional solutions are required to improve accuracy.

To increase accuracy, we set the location beacon’s broadcasting cycle as 0.1 s while updating the indoor map every 1 s. Theoretically, 10 RSSI values can be obtained in such a cycle and each location can be estimated as well. The method that designates the average of 10 estimated locations as the final estimated location was used here but the accuracy had dropped to 60% as the performance time of the Bluetooth scanning module was over 0.1 s so that only about 4 to 6 RSSI values were collected. For this reason, it became more difficult to acquire synchronized RSSI values between location beacons. We were able to confirm that the accuracy can be increased by setting a shorter updating cycle.

### 4.2. Improvement of Performance of Smart Phone Gyroscope (Scenario 2)

**Figure 15:**
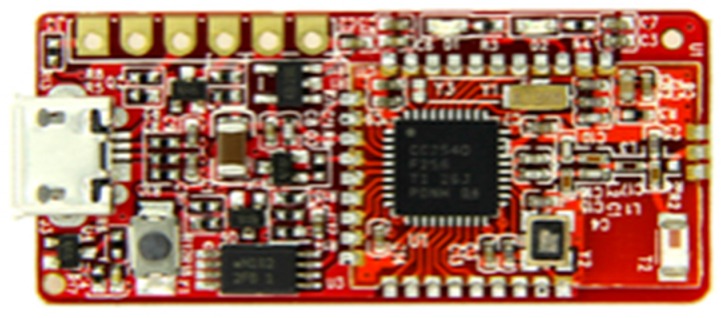
TI CC2540 module (Using Scenario 2).

Firmware has been compiled and installed in a ROM so that the information can be broadcasted in accordance with the iBeacon standard to measure RSSI periodically. Figure 15 shows an image of the TI CC2540 Module [9].

#### 4.2.1. Position Measuring Android Application

For the development of the position measuring application, the range size was set to 20 and the threshold was set to 7.5 in a range-average for the trend estimation after a small-scale preliminary test to measure RSSI. The RSSI sampling was carried out by collecting samples every 100 ms while shifting the distances from 1 m to 3 m for 70 s and the same process was repeated multiple times [9].

**Figure 16:**
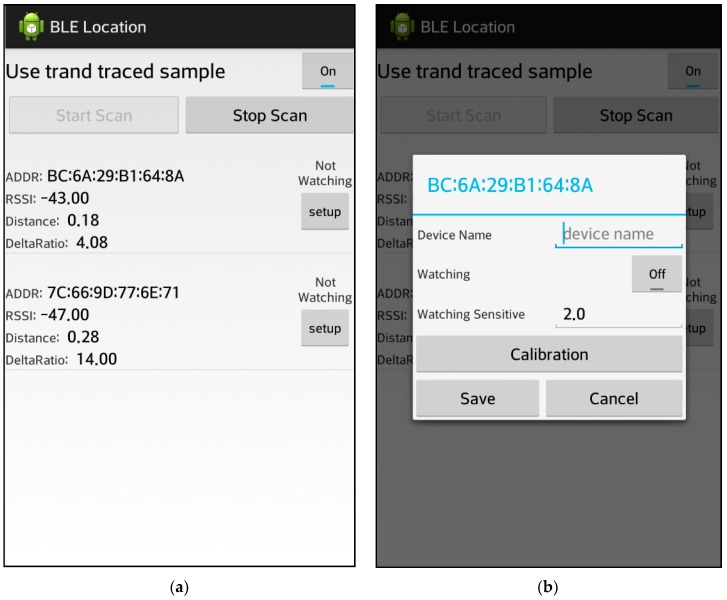
Position measuring application (Scenario 2). (**a**) User Interface (1); (**b**) User Interface (1).

Figure 16a shows an interface for the application and the scenario in which a distance is estimated using the TI CC2540 module connected via Bluetooth using the RSSI value and the deltaRatio. Figure 16b shown an interface where the connection has been established between the terminal that is using the same module.

#### 4.2.2. Motion (Shifting) Vector Extraction Using an Accelerometer

**Figure 17:**
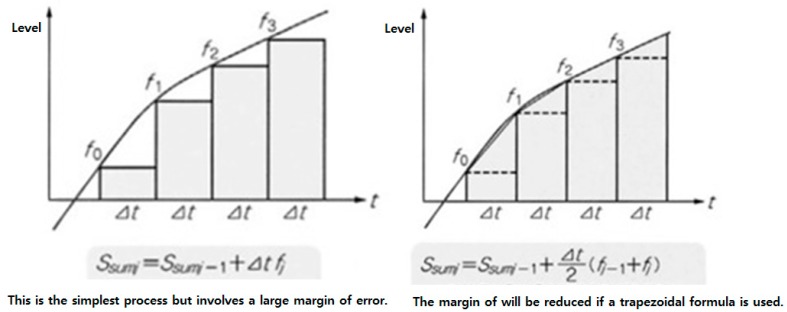
Trapezoid integration.

As shown in Figure 17, to extract a motion vector using an accelerometer, the speed can be obtained by integrating the accelerations and the distance can be estimated by integrating the speeds. Then, a three-dimensional motion vector was extracted by performing the double integration for length, width and height, respectively. Since integration errors may result from an ordinary distinction, a primarily reinforced trapezoid integration was used.

#### 4.2.3. Removing Gravitational Acceleration

**Figure 18:**
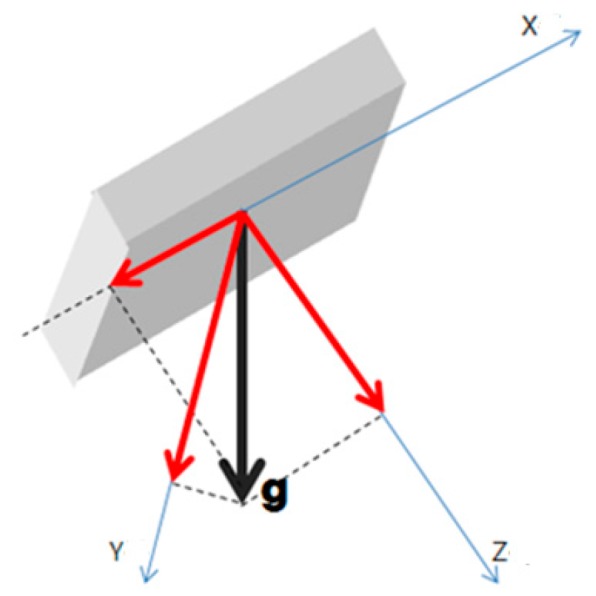
A method of measuring position of equipment.

As shown in Figure 18, the value measured by the accelerometer will be affected by the gravitational acceleration. The position of the equipment was measured with a gyroscope and the impact of the gravitational acceleration was estimated.

#### 4.2.4. An Android Application Measuring the Distance between the Beacons

**Figure 19:**
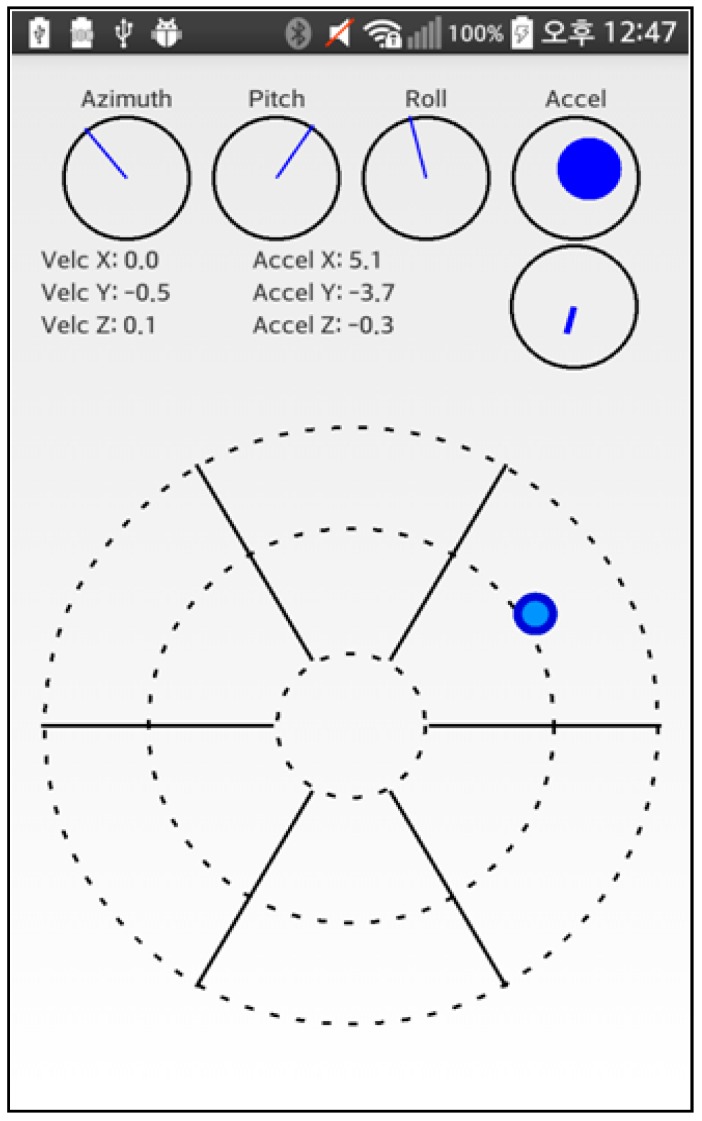
An Android application that measures distance to a Beacon (Scenario 2).

A three-axis value measured with a gyroscope has been visualized as well as the current acceleration level and motion vector, together with corresponding values. By placing the measuring equipment at the center, a beacon could be placed in the estimated direction and the rough distance could be calculated. Figure 19 shows an Android application that measures the distance to a beacon.

#### 4.2.5. Development of a Sampling Method for Removing RSSI Noises

**Figure 20:**
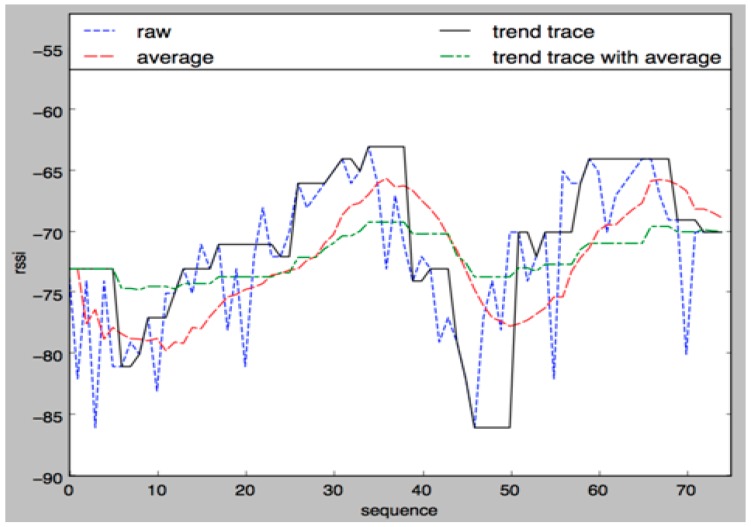
Signal changes after using a smooth filter.

Figure 20 shows the signal changes that occur while using a smooth filter. Primarily, the overall trend of signal changes was extracted using a filter and the consistency of recently measured samples with the extracted trend was checked afterwards. The noise-resistant and trend-sensitive characteristics are shown in black and the values that were consistent and will be used in an actual application are indicated with the green dotted line.

To test whether the bearing of the beacon can be estimated by measuring the motion (movement) of the measuring equipment, the distances for the measurement were 1 m to 3 m, which moved away and came closer within the duration of one minute. This process was repeated 10 times and measurements were taken.

#### 4.2.6. Classification by Initialization Method

(a) Measurement by Initialization (The Same Direction)

Measurements were taken by setting the initial value of discovered beacon’s that were identical to the actual position of the beacon.

(b) Measurement by Initialization (Orthogonal Direction)

This measurement was taken by setting the initial value of discovered beacon’s bearing opposite to the actual position of the beacon.

#### 4.2.7. Classification of Motions

(a) Moving the Shortest Distance

Moving in the forward direction of the intersecting line between the beacon and the measuring equipment.

(b) Orthogonal Movement

Moving in the orthogonal direction of the intersecting line between the beacon and the measuring equipment.

(c) Measurement by Rotation

After setting the direction of the beacon, the difference between angles (i.e., between the actual angle of a beacon and software-calculated angle) was measured to determine whether the position of the beacon was recorded by the measuring equipment when it was rotated horizontally with the ground. The position was initialized and the margin of error was calculated after rotation was carried out 20 times.

#### 4.2.8. Performance Analysis

Measurements were taken five times using this measuring method and then a mean Scala value was used. The results are provided in Table 6.

Measurements were taken five times using this measuring method and then a mean Scala value was used. As a result, the error rate was 4.36. The margin of error will be small if initialization is carried out properly and the movement is made forward but if the initialization is incorrectly performed, much time will be required to correct the involved error. Thus, improving this situation will be a future task.

#### 4.2.9. Proposition

For the beacon position estimation, there are many cases of incorrect shifting distance measurements when the measured value of deceleration range is smaller than that of acceleration range due to lack of precision in an accelerometer and this makes calibration difficult. From a long-term perspective, additional developments will be needed for the filters that can reduce or remove such a situation. The variation in the above-mentioned measuring methods (by forward and orthogonal movements) was quite large and needs improvement. Although the proposed algorithm was developed to be used in a wide space, the test bed in such a space was never achieved. It is author’s intention to schedule this task after applying for a patent and proceeding with commercialization.

## 5. Conclusion and Future Works

An indoor location-based control system that provides services by estimating user’s indoor locations has been implemented in this study (First scenario). The system consists of a localization server, service-provision client and user application. The server estimates the location by using trilateration and COG calculation for the hexagonal indoor spaces. The service-provision client is an element that provides services based on the location information acquired with the webcam-based indoor monitoring program. The user application delivers the RSSI data to the server.

By integrating communication technology with Bluetooth Beacons and the proposed system, a real time service similar to that offered by blackboxes can be provided to users so that it will be helpful in avoiding disputes over navigation service or cost savings.

A method that facilitates system extension in various indoor spaces by partitioning an indoor space into several hexagonal basic unit spaces was proposed as well. For the estimation of indoor locations, the trilateration and COG calculation based on the RSSIs of the Bluetooth signals in a WLAN environment were used. The characteristics of wireless signals were studied, followed by investigation of causes of inaccurate location estimations. The key to a robust localization system is the accuracy so that we have proposed a method that selects the target of trilateration within the hexagonal basic unit space to increase the accuracy. However, we found that the methods proposed through the experiments conducted here were insufficient to provide useful services as they did not provide an adequate level of accuracy. The implemented system provided an accuracy level of approx. 74% when the margin of error was 1 m. The other 14% were found to be far apart from the actual locations such that the accuracy can be improved up to 88% if the system can estimate locations more precisely. Therefore, we propose using the technology based on the cumulative probability distribution. It is expected that the locations will converge to exact coordinates as the indoor location data piles up, dismissing the distant coordinates. The indoor location-based control systems with an increased accuracy will provide more useful services to the users. Providing an indoor navigation service to the people who cannot acquire any visual information due to visual impairment can be a good example.

For estimating beacon position, there are many cases of incorrect shifting distance measurements when the measured value of the deceleration range is smaller than that of the acceleration range due to a lack of precision in an accelerometer and this makes calibration difficult. In the long-term perspective, additional developments for filters that can reduce or remove this type of occurrence will be needed. The variation in the above-mentioned measuring methods (by forward and orthogonal movements) was quite large and needs improvement. Although our proposed algorithm was developed to be used in a wide space, the test bed in such a space was never achieved. It is the author’s intention to schedule this task after applying for a patent and proceeding with commercialization.

The algorithm introduced in this study (Second scenario) is effective in extracting valid samples from the RSSI dataset but has it has some drawbacks as well. Although we used a range-average algorithm that measures the shortest distance, there are some limitations because the measurement results depend on the sample size and the sample efficiency depends on sampling speeds and environmental changes. Thus, further research is needed to obtain more precise results and to find a better algorithm that is adaptable in each different environment.

Additionally, even if the trend changes due to rapid shifts in the distance measuring equipment, the changes can be anticipated by presenting a low-precision measurement value. While the noises will largely affect results when threshold values are too big, lower reactivity can be expected if they are too small. Future research in this area should take this into consideration.

With the Delta-based RSSI sampling method and its resulting samples, the degree of precision can be increased, which is why we looked into this method as one of the feasible options for developing IoT-based technologies for indoor positioning systems. Another element to consider is power consumption rate and we used the BLE-exclusive beacons to keep the rate lower. The beacons can last up to 1.8–28.7 months with a CR2045 battery. It is difficult to use a positioning system like GPS indoors. Thus, using the BLE-based indoor positioning system in department stores, large buildings and subways could be a possibility and the system’s application areas will be increased. The Bluetooth system can be implemented at a relatively low cost so that once the problem of precision is solved, it can be applied to various fields.

**Figure 21:**
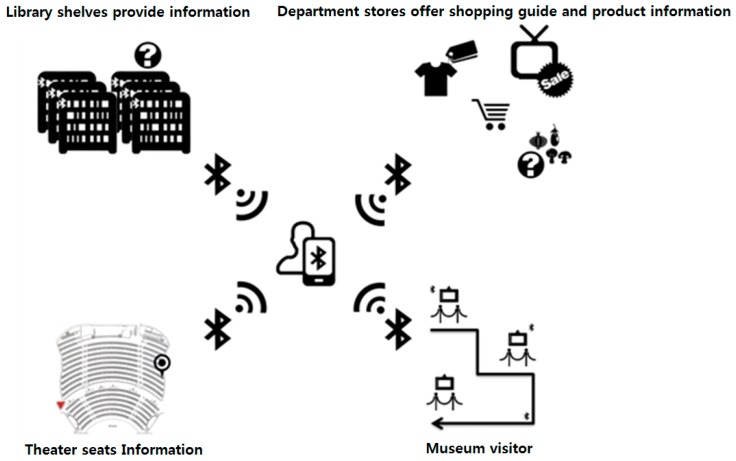
A concept diagram of the indoor positioning system application.

The concept diagram in Figure 21 shows the change in distance between the reference measuring equipment and the BLE devices, so that if BLE devices are embedded in products, the system will be useful in various places (e.g., museums, exhibitions, etc.) as an alarm system.

In the initial stage of our study, it was to track the position by using multiple beacons for triangulation purpose, the method has been changed to tracking the bearings of a single beacon. As a result, the system is simpler in its organization and it was possible to construct one that was more convenient and useful in most indoor environments. Another consideration would be improving the accumulation/integration algorithm for the estimated bearings as the results could reveal a larger margin of error depending on the shifts or intersections of beacons and measuring equipment.

## Figures and Tables

**Table 1:** Signal attenuation constants measured in an experimental space.

Distance (m)	N	RSSI (dBm)
1	−	59
2	3.322	70
3	4.610	82
*Tx* of the Bluetooth module M-10 is 59		

**Table 2:** Commands for client.

Command	Description	Example
IDEN	Informs client’s unique ID to the server when accessing it	IDEN id
RSSI	Transmits RSSI values of location beacons to the server	RSSI minorA: rssiA
LIST	Requests an indoor map to the server	LIST
LOCT	Requests the location of a client relevant to the ID	LOCT id

**Table 3:** Commands for server.

Command	Description	Example
IDEN	Informs an access state to the client	IDEN OK
		IDEN OVERLAP
RSSI	Acknowledges reception of a RSSI value	RSSI OK
		RSSI ERROR
LIST	Delivers the indoor map	LIST id; x, y, area
		LIST NOONE
		LIST ERROR
LOCT	Provides the location of a client relevant to the ID	LOCT x:y:area

**Table 4:** List of equipment used for the experiments.

Equipment	Function	Description	Number
HM-10	Location beacon	Broadcasts a Bluetooth signal every 0.546 s with the Bluetooth module	7
		Low-power operation is enabled with Bluetooth 4.0 technology	
Laptop	Localization server	Executes the program written with the Python language	1
		The server updates the indoor map by acquiring the RSSI values from the user application and provides the map to the service client	
	Camera-based monitoring client	Executes the client program written with the Java Swing	1
		Connected with webcam, the program provides the indoor surveillance service by acquiring the indoor map form the localization server	
Android Smart Phone	User application	Galaxy S6 Edge Smart phone with the OS Android Marshmallow (API level 23) was used for the experiment	1
		User application acquires the RSSI values every 0.546 s by scanning the signals from the location beacons and transmits them to the server	
		If not the latest model, it will take much more time to scan the signals	
Wireless Router	Private network	Assumes the role of the DHCP (Dynamic Host Configuration Protocol) server. Assigns IPs to the communication devices and perform communications relay between server and client	1

**Table 5:** HM-10 activation commands by HM-10.

Command	Description
AT + RENEW	Initializes HM-10
AT + RESET	Performs rebooting
AT + MARJ	Sets major for iBeacon (Cf. Section 3.1.2)
AT + MINO	Sets minor for iBeacon
AT + ADVI5	Sets signal advertising cycle AT + ADVI5 refers to 0.546 s
AT + ADTY	Sets non-connectable mode
AT + IBEA1	Activates iBeacon
AT + DELO2	Sets broadcast-only mode
AT + PWRM0	Sets auto-sleep mode (Min. power use mode)
AT + RESET	Reflects the settings through rebooting

**Table 6:** Performance evaluation for motion measurement.

Classification (Degree)	Same Direction Initialization	Orthogonal Direction
Shortest Distance Shifting	6.47	18.94
Orthogonal Direction Shifting	23.79	42.13
